# An Organ System-Based Synopsis of *Pseudomonas aeruginosa* Virulence

**DOI:** 10.1080/21505594.2021.1926408

**Published:** 2021-06-27

**Authors:** Charles D. Morin, Eric Déziel, Jeff Gauthier, Roger C. Levesque, Gee W. Lau

**Affiliations:** a Centre Armand-Frappier Santé Biotechnologie, Institut National De La Recherche Scientifique (INRS), Laval, Quebec, Canada; b Département De Microbiologie-infectiologie Et Immunologie, Institut De Biologie Intégrative Et Des Systèmes (IBIS), Université Laval, Québec City, Quebec, Canada; c Department of Pathobiology, University of Illinois at Urbana-Champaign, Urbana, IL, US

## Abstract

Driven in part by its metabolic versatility, high intrinsic antibiotic resistance, and a large repertoire of virulence factors, *Pseudomonas aeruginosa* is expertly adapted to thrive in a wide variety of environments, and in the process, making it a notorious opportunistic pathogen. Apart from the extensively studied chronic infection in the lungs of people with cystic fibrosis (CF), *P. aeruginosa* also causes multiple serious infections encompassing essentially all organs of the human body, among others, lung infection in patients with chronic obstructive pulmonary disease, primary ciliary dyskinesia and ventilator-associated pneumonia; bacteremia and sepsis; soft tissue infection in burns, open wounds and postsurgery patients; urinary tract infection; diabetic foot ulcers; chronic suppurative otitis media and otitis externa; and keratitis associated with extended contact lens use. Although well characterized in the context of CF, pathogenic processes mediated by various *P. aeruginosa* virulence factors in other organ systems remain poorly understood. In this review, we use an organ system-based approach to provide a synopsis of disease mechanisms exerted by *P. aeruginosa* virulence determinants that contribute to its success as a versatile pathogen.

## INTRODUCTION

**Figure 1.**
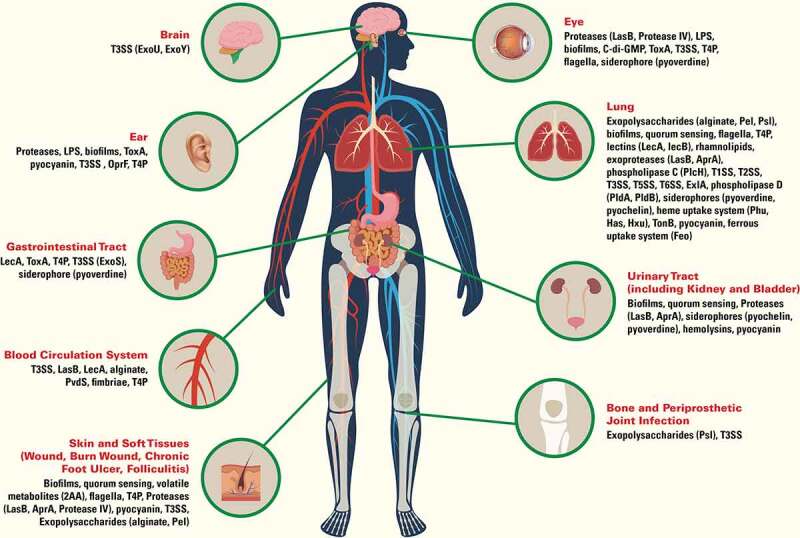
Virulence factors of *Pseudomonas aeruginosa* important for infection in human organs. Type 1 secretion system (T1SS), type 2 secretion system (T2SS), type 3 secretion system (T3SS), type 5 secretion system (T5SS), type 6 secretion system (T6SS), lipopolysaccharides (LPS), exotoxin A (ToxA), outer membrane protein F (OprF), Type IV pili (T4P), lectin A (LecA), lectin B (LecB), elastase (LasB), alkaline protease (AprA), phospholipase C (PlcH), 2-aminoacetophenone (2AA), cyclic diguanylate (c-di-GMP), exolysin (ExlA), phospholipase D (PldA, PldB)

*Pseudomonas aeruginosa* is a ubiquitous Gram-negative bacterium found in soil and surfaces of aqueous environments. Because of its metabolic versatility and high intrinsic resistance to antimicrobials, *P. aeruginosa* efficiently adapts and thrives in a wide variety of natural and artificial settings, including in-hospital facilities and patient devices. Although rarely infecting healthy individuals, *P. aeruginosa* is a leading and notorious opportunistic pathogen [1,2], especially in immunocompromised patients with defective immune defenses, including chronic neutropenia and defects of neutrophil function, cancers, human immunodeficiency (HIV) acquired immunodeficiency syndrome (AIDS), and diabetes mellitus. *P. aeruginosa* is best known to chronically colonize and infect the lung of people with cystic fibrosis (CF) and advanced stages of chronic obstructive pulmonary disease (COPD) [3,4]. Also, *P. aeruginosa* is responsible for approximately 4% total cases of hospital-acquired bloodstream infections, and was the third leading cause among Gram-negative pathogens [5]. Nosocomial pneumonia, especially the ventilator-associated pneumonia (VAP) is a major cause of morbidity and mortality in critically ill patients, and the isolation of *P. aeruginosa* is associated with worse clinical outcomes [6,7]. *P. aeruginosa* is also known to cause a wide variety of other infections, encompassing all organs of the human body, including soft tissue infection in burns, open wounds and postsurgery; urinary tract infection associated with the use of urinary catheter; foot infection in diabetics and individuals with impaired microvascular circulation; ear infection, especially otitis externa and chronic suppurative otitis media associated with tissue injury and water blockage; and keratitis associated with extended contact lens wear and contaminated contact lens [8]. Other rarer but serious infections include endocarditis occurring in patients with or without injection drug use [9,10]; and meningitis associated with penetrating trauma to the head, placement of a CNS shunt (such as a ventriculoperitoneal (VP) shunt), or post-neurosurgical procedures [10,11]. Poor antibiotic stewardship in the past few decades has led to emergence and spread of multidrug-resistant strains. Effective treatment options are increasingly scarce; consequently, *P. aeruginosa* infections are associated with high morbidity and mortality. Not surprisingly, carbapenem-resistant *P. aeruginosa* is classified as a serious threat by the Centers for Disease Control and Prevention of the United State (US CDC, https://www.cdc.gov/drugresistance/biggest-threats.html), and is on the World Health Organization (WHO) Priority 1 list of pathogens for research and development of new antibiotics (https://www.who.int/medicines/publications/WHO-PPL-Short_Summary_25Feb-ET_NM_WHO.pdf). In this review, we use an organ system-based approach to provide a brief overview of pathogenic mechanisms exerted by *P. aeruginosa* virulence factors that contribute to its success as a versatile pathogen (Figure 1).

## VIRULENCE FACTORS

This section presents a brief summary of the main virulence factors of *P. aeruginosa* and their mechanisms of action. Detailed function of these virulence factors specific to individual organ systems are presented in their respective sections.

### Biofilm formation

Establishment of a microbial biofilm relies on the formation of a matrix composed of extracellular polymeric substances that embeds the bacteria together into a robust colony [*P. aeruginosa* biofilm formation has been reviewed in details here [12]]. The production of this matrix allows for long-term persistence of *P. aeruginosa* on biotic and abiotic surfaces by shielding the population from antimicrobial agents and adverse conditions. Three exopolysaccharides – Pel, Psl and alginate – along with extracellular DNA and proteins constitute the bulk of the exopolymeric substances forming the matrix. These bacterial biofilm communities are typically refractory to antibiotic therapy and resistant to host immunity, and are therefore, difficult to treat.

### Exoproducts and secretion systems

*P. aeruginosa* secretes a multitude of toxic metabolites, often through protein complexes called secretion systems (reviewed in the context of *P. aeruginosa* [13]; all known families of secretion systems [14]). The Type 1 secretion system (T1SS) exports the alkaline protease AprA. The Type 2 secretion system (T2SS) is responsible for the secretion of multiple virulence factors, notably: exotoxin A (ToxA), proteases LasA and LasB, and the hemolytic phospholipase C (PlcH) [13]. The Type 3 secretion system (T3SS) is a needlelike nanomachine that delivers toxic effectors – ExoS, ExoT, ExoU and ExoY – into the target host cell (reviewed in [15]). Additionally, a subgroup of T3SS-deficient *P. aeruginosa* clinical isolates cause hemorrhagic pneumonia by secreting the exolysin ExlA through the Type 5 secretion system (T5SS) [16]. The Type 6 secretion system (T6SS) apparatus is mostly involved in secretion of effectors for interbacterial killing, although some T6SS effectors target eukaryotic hosts [17].

Pyocyanin is one of the phenazines secreted by *P. aeruginosa* that imparts a blue color to many abscesses and burn wounds infected by the pathogen. Among the phenazines, pyocyanin is best studied, and is considered most toxic because of its redox-active and zwitterionic properties. Pyocyanin easily traverses the cell membrane and causes oxidative stress by generating reactive oxygen and nitrogen species (ROS and RNS), which allow *P. aeruginosa* to kill competitor microbes inhabiting the same niche, as well as damaging host cells or modulating their immune signaling [18].

*P. aeruginosa* also produces rhamnolipids (RLs), a class of amphiphilic glycolipids with surface-active properties [19]. Despite the immense interest toward RLs as an ecological alternative to synthetic surfactants [20], their role in *P. aeruginosa* virulence has been proportionally underappreciated.

### Quorum sensing

“Quorum sensing” (QS) is the archetypal intercellular signaling system used by many bacteria to regulate their gene expression co-ordinately and synergistically as a group [21]. *N*-acyl-L-homoserine lactones (AHLs) are the signals mediating the well-characterized QS system used by many Gram-negative bacteria [21,22]. The complex QS regulatory pathway in *P. aeruginosa* controls the expression of multiple virulence factors (recently reviewed in [23,24]). In this species, two separate transcriptional regulator/autoinducer synthase pairs, LasR/LasI and RhlR/RhlI, modulate the transcription of target genes in response to their respective cognate AHLs, oxo-C_12_-HSL and C_4_-HSL [25]. *P. aeruginosa* also produces QS signals distinct from AHLs, the 4-hydroxy-2-alkylquinolines (HAQs)[26], such as 3,4-dihydroxy-2-heptylquinoline, the *Pseudomonas* Quinolone Signal (PQS) [27]. The transcriptional activator MvfR (aka PqsR) controls the biosynthesis of HAQs [26] by the *pqsABCDE* operon [26,28,29]. The enzyme PqsH [28,30] is also required for synthesis of PQS, the main autoinducer of MvfR [29,31]. All three QS systems play a vital role in *P. aeruginosa* pathogenesis [32–34], upregulating (directly or indirectly) the transcription of over 300 genes [35–37], many encoding virulence functions [32,38], including the elastase LasB [39], hydrogen cyanide, phenazines such as pyocyanin [40], RLs [41], biofilm development [42] and two of the T6SSs [43,44].

### Iron acquisition systems

Since iron is a limiting microelement in the environment because of its low solubility in the ferric form (Fe^3+^), *P. aeruginosa* evolved multiple molecular mechanisms to scavenge this essential but scarce resource (reviewed in [45]). Two iron-binding siderophores, pyoverdine and pyochelin, are secreted. These siderophores have high and low affinities for ferric iron, respectively, and once iron-bound they can be imported back into the cell. When ferrous iron (Fe^2+^) is available, for instance, under anaerobic conditions or following reduction by phenazines, *P. aeruginosa* utilizes its Fe^2+^ uptake system FeoABC. *P.aeruginosa* also possesses three heme import systems to acquire iron from the host hemoproteins, including the Phu (*Pseudomonas* heme uptake) and the Has (heme assimilation system), and the recently discovered Hxu system [46].

### Cellular appendages and adhesins

*P. aeruginosa* possesses various means of motility and attachment, namely a polar flagellum (reviewed in [47]), multiple types of type IV pili (T4P: type IVa, IVb pili, tight adherence pili; all reviewed in [48]), chaperone-usher-pathway (cup) fimbriae (reviewed in [49]), and other adhesins such as the two carbohydrate-specific lectins: PA-IL (also named LecA) and PA-IIL (also named LecB) (reviewed in [50]). The lipopolysaccharide (LPS) is a constituent of the external layer of the outer membrane comprised of lipophilic anchor lipid A, an inner oligosaccharide core and an outer oligosaccharide chain, the O-antigen [51]. The presence of these appendages on the bacterial cell surface means that they are recognized by the immune system of the host.

## LUNG INFECTION

The human respiratory system is divided into the upper and lower tracts. The upper tract is composed of nasal cavity, pharynx and larynx whereas the lower tract includes the trachea, primary, secondary and tertiary bronchi, bronchioles and ≥ 700 millions alveoli with a mesh-like network of tiny capillaries and venules where gas exchanges occur. Apart from gas exchanges, the respiratory system as a whole is also involved in sensing the environment, secretion, regeneration, preventing infections, processing toxins and removing debris. Airway secretory cells produce mucins and antimicrobial peptides and metabolize toxins, whereas ciliated cells use their cilia to propel debris out of the lung [52]. A recent single-cell RNA sequencing analysis of approximately 75,000 cells spanning the entire lung compartments and circulating blood has further defined the gene expression profiles and anatomical locations of 58 cell populations within the human lung, including 14 uncharacterized cell types. This molecular atlas provides novel insights into the functions, regulation and interactions of the known and new cell types, and will likely engender major advances in our understanding of pulmonary biology and the interactions between lung and *P. aeruginosa* and other respiratory pathogens[53^53^].

The human lung presents a challenging immunological dilemma for the host. While simultaneously facilitating vital gas exchanges, it is constantly bombarded by chemicals, pollutants and microbes inhaled from the environment [54]. The epithelial surface of healthy airways is covered by the airway surface liquid (ASL), which is comprised, among others, of mucins, antimicrobial peptides and proteins, innate immune cells, and signaling molecules. The bilayer ASL contains the periciliary layer sandwiched between the top mucus gel layer and the bottom airway epithelium, forming the so called “gel-on-brush” structure [55]. Hydrogel within the periciliary layer provides space for ciliary beating and supports mucociliary clearance. The predominant mucins MUC5AC and MUC5B provide viscosity and gel-forming properties to mucus, which traps inhaled pathogens and irritants, keeps moisture in the airway epithelium [56], and helps to maintain the periciliary layer and mucociliary clearance. Both MUC5AC [57] and MUC5B [58] are critical for innate immune defense of the airways against microbial infection. However, excessive mucus and failure in clearance by the mucociliary escalator will clog the airways, which will then reduce and prevent air flow into the alveolar space. This is a common pathological condition found in multiple lung diseases such as cystic fibrosis (CF), chronic obstructive pulmonary disease (COPD), asthma, primary ciliary dyskinesia, chronic bronchitis and VAP. Excessive mucus creates a niche for microbial colonization, which will exacerbate vicious cycles of proinflammatory response, lung damage, and morbidity and mortality [59–62].

Two of the best-studied chronic lung diseases where microbial infections play a critical role in disease pathogenesis are CF and COPD. CF is a fatal genetic disorder affecting approximately 70,000 individuals world-wide. Although most commonly found in the Caucasian population, with 1 in 2,000 to 3,500 cases, CF is also found among other ethnic populations, at 1 in 16,000 to 17,000 among Africans, and at 1 in 30,000 among Asians. CF is caused by mutations in the **c**ystic **f**ibrosis **t**ransmembrane **c**onductance **r**egulator (CFTR) gene, which encodes a chloride (Cl^−^) channel. The loss of CFTR function reduces Cl^−^ secretion, causing an increased transepithelial absorption of sodium and water. This results in thickening of the mucus in the airways, which slows down and disables the mucociliary escalator function, creating a suitable niche for microorganisms to thrive [4,63,64]. The major clinical problem for CF patients is progressive loss of pulmonary function caused by chronic lung infection with *P. aeruginosa*, which causes morbidity and mortality, with an average life expectancy in the 40s. Repeated bouts of inflammation triggered by the bacterium progressively damage the lung. Although antibiotics can decrease the frequency and duration of acute infectious exacerbations, this opportunistic pathogen establishes permanent residence and is never completely eradicated from lungs. COPD is a leading cause of morbidity and mortality worldwide, affecting 329 million people and cause over 3 million deaths annually [65,66]. The odds of developing COPD are increased by cigarette smoking, exposure to air pollutants, biomass smoke (e.g., indoor wood burning), and toxic desert dusts inhaled by soldiers in the Middle East [65–69]. Long-term exposure to these irritants exacerbates the inflammatory response, narrows the small airways, and causes emphysema. The most common symptoms of COPD are excessive mucus and sputum, shortness of breath, chest tightness and wheezing. The majority of cases of acute exacerbation in COPD are caused by microbial infections [70,71], among which, *P. aeruginosa* is a common cause of severe exacerbations, especially in advanced stages of COPD [3,72,73].

Because *P. aeruginosa* is metabolically versatile, possesses high intrinsic antibiotic resistance, and expresses a large repertoire of virulence factors, it is proficiently adapted to thrive in a wide variety of environments, and in the process, making it a notorious opportunistic pathogen in the complex proinflammatory milieu within chronically diseased lungs such as CF and COPD. Most of our understanding of *P. aeruginosa* pathogenesis is derived from the context of CF. Various virulence factors that drive lung colonization and infection are briefly summarized below.

## EXOPOLYSACCHARIDES AND BIOFILMS

### Biofilms

Biofilm formation plays key roles in the persistence of *P. aeruginosa* in chronically diseased lungs, especially in CF (reviewed in [74]). Long-term growth in the CF lung environment gives rise to different phenotypic variants of *P. aeruginosa*, two of which exhibit enhanced biofilm-producing phenotypes: mucoid variants and small colony variants (SCVs) [75,76].

### Exopolysaccharides alginate, Pel and Psl

Mucoidy results from the overproduction of alginate, due to a mutation in the gene coding for the anti-sigma factor MucA[75]. A long-term study of 56 CF patients revealed a median time of about 11 years for the transition of non-mucoid *P. aeruginosa* (normally acquired in the first years of life) to a mucoid variant, a transition followed with worsening symptoms [77]. Production of alginate has been linked to increased resistance to host-produced antimicrobial peptides, phagocytosis by leukocytes and antibiotics [78–81]. Despite their apparent benefits, mucoid isolates are often present as mixed populations with their non-mucoid counterpart, the latter often being the product of phenotypic reversion due to suppressor mutations in *algT* [82,83]. Such mixed population might be at an advantage, as Malhotra *et al*. (2018) demonstrated that a mixed population of mucoid and non-mucoid variants is more resistant to both antimicrobial peptides (conferred by mucoid members of the population) and H_2_O_2_ (resistance granted by the non-mucoid subpopulation) [81]. A recent study assessing the distribution of mucoid and non-mucoid isolates from bronchoalveolar lavages of specific lobar regions of the lungs confirmed that mucoid variants do not preferentially localize to certain lobes, and that their presence is marked by elevated levels of proinflammatory cytokines in colonized regions[82]. Alginate might also play an important role in the context of mixed bacterial infections in the CF lungs. Alginate leads to reduced killing of *Staphylococcus aureus* by *P. aeruginosa* in an *in vitro* co-culture [84]. The same study showed that this protective effect can be achieved simply by adding exogenous alginate to a co-culture with an alginate biosynthesis deficient mutant of *P. aeruginosa*. Exogenous alginate increased the persistence of *Burkholderia cenocepacia, B. multivorans, Haemophilus influenzae*, but not *Stenotrophomonas maltophilia* and *B. cepacia*, in the lungs of a CFTR knockout mouse [85]. These six other bacterial pathogens are also prevalent in the CF lungs [86].

Like their name suggests, SCVs appear as smaller colonies when grown on an agar surface (reviewed in [87]). The emergence of a subpopulation of SCVs is a frequently observed feature of *P. aeruginosa* isolates from CF lungs biofilms [76,88]. The first characterization of SCVs from CF patients highlighted a two to eight-fold increase in resistance to antibiotics [89]. This same study also indicated that SCVs are found more predominantly in patients undergoing aerosolized antibiotics therapy in contrast to those with intravenous therapy. These results suggest that antibiotic aerosolization leads to the emergence of drug tolerance in SCVs. The presence of SCV is also correlated with severe impairment of lung function [89]. Like the mucoid variants, SCVs promote persistence in the CF lungs as they possess higher resistance to neutrophil antimicrobials such as H_2_O_2_ and antimicrobial peptides and cause more inflammation [90].

The importance of biofilms in pulmonary infections is also highlighted in a study where transcriptomics were used to compare transcript levels between multiple CF sputum samples and lab-grown clinical isolates were compared[91]. Among the key differences between both groups of samples, there was a marked increase in the transcript levels of Pel, Psl and alginate biosynthesis pathways in CF sputum samples compared to lab-grown isolates [91]. Both Pel and Psl, but not extracellular DNA, were found to play an important role in the *in vitro* formation of biofilm by mucoid *P. aeruginosa*; both exopolysaccharides also contributed to the resistance of the same mucoid strain against host defense in a mouse model of acute pneumonia [92].

*P. aeruginosa* biofilms also play a role in the pathogenesis of VAP (reviewed in [93]). Biofilms will form on the endotracheal tube upon intubation of a patient, providing a direct access to the lower respiratory tract [94]. A study compared the *in vitro* biofilm formation capabilities of clinical isolates from mechanically ventilated patients and CF patients, and found that all isolates from intubated patients produced more biofilm in a 96-well plate assay than both CF isolates and the reference strain PAO1 [95]. Although not fully understood, these VAP strains are likely better adapted to form biofilms on rough and porous plastic surfaces of microtiter plates and medical devices. Additionally, these authors also found that strains which produce more biofilms are positively correlated to heightened antibiotic resistance, particularly to β-lactam antibiotics, suggesting a selection pressure to hyper biofilms producing phenotype.

## QUORUM SENSING

QS mutants of *P. aeruginosa* are attenuated in their ability to cause mortality in various hosts [96–101]. *P. aeruginosa* mutants lacking QS regulatory genes cause less lung pathology in mouse and rat models, suggesting that cell-to-cell signaling plays a key role in virulence [97,98,101]. For instance, a *lasR-* mutant was virtually avirulent in a neonatal mouse model of pulmonary infection compared to pneumonia caused by the parental wild type strain PAO1 [102]. In addition, sputum samples and lung exudates from CF patients chronically infected with *P. aeruginosa* contain significant amounts of AHLs and PQS, indicating that all three QS systems are functioning during human infection [103,104].

It is noteworthy that *P. aeruginosa* isolates from chronically infected CF patient lungs, but not from acute infections, frequently contain *lasR* mutations. [105–107] Loss of LasR function occurs over the years in these chronically colonized individuals, indicating strong selective pressure against LasR activity [107–109].; a marker of poor prognosis, these mutants are associated with progression of the CF disease [106]. Interestingly, Feltner *et al.* (2016) have shown that some LasR-deficient variants isolated from CF lungs have maintained a QS-dependent virulence gene expression, through RhlR activity [110]. In these isolates, the authors noted that “*there is apparently no correlation between the absence of a functional LasR and a variety of quorum-regulated phenotypes”.*

Multiple studies have demonstrated that AHLs, especially oxo-C_12_-HSL, are able to modulate host immune functions, with either detrimental or beneficial effects [reviewed here [111,112]]. Oxo-C_12_-HSL induces the chemotaxis of neutrophils, and increases their phagocytic capacity, which could be due to modulation of (CD)11b/CD18 integrins and CD16 and CD64 immunoglobulin receptors [113]. Similarly, oxo-C_12_-HSL stimulates phagocytic activity of human macrophages by inducing the p38 MAPK, which causes positive changes in cell volume, morphology, and water channel AQP9 that promotes phagocytosis [114]. In contrast, oxo-C_12_-HSL, but not C_4_-HSL, induces apoptosis in phagocytic cells [115], interferes with proper functioning of mitochondria [116], and alters the expression and secretion of multiple pro- and anti-inflammatory cytokines and chemokines [117,118]. However, these results need to be interpreted with caution because the vast majority of host modulation studies were performed *in vitro* using oxo-C_12_-HSL in concentrations between 300 and 600 μM, often in different immortalized cell lines. In contrast, only low μM concentrations of C_4_-HSL and low nM concentrations of C_12_-HSL have been detected in CF lungs [103,104,119,120]. Nevertheless, it is possible that during biofilm-mediated infection, locally-enhanced concentrations of QS molecules and virulence factors could modulate host immune functions.

## CELL APPENDAGES AND ADHESINS

### Flagellum

As presented above, *P. aeruginosa* uses various mechanisms for attachment and motility. A *P. aeruginosa* mutant that lacks its flagellar fiber is more sensitive to the membrane permeabilizing activity of the pulmonary surfactant protein A. This protective effect was linked in part to a lack of O-antigen in the outer membrane of flagellum-null mutants and reduced expression of secreted proteases which would degrade the surfactant protein A [121,122]. In a mouse model of acute pneumonia, a non-lethal dose of *P. aeruginosa* expressing its flagellin fiber was cleared more rapidly by the host, while challenge with a lethal dose (equivalent to the LD_50_) led to more rapid killing of mice, compared to mutants lacking the flagellum [123]. The flagellin acts as an adhesin capable of binding to the mucin Muc1 when expressed in the CHO cell line [124].

### Type IV pili

*P. aeruginosa* mutants lacking either *pilT* and *pilU*, both encoding proteins responsible for the retraction of the T4P, still express surface T4P but show reduced cytotoxicity against lung pneumocytes, lower cell adhesion and attenuated virulence in a mouse model of acute pneumonia [125]. In the same study, a pilin (*pilA*)-null mutant showed an even more pronounced reduction in virulence [125]. Similar to flagellin, the presence of T4P at the surface of the cell also confers a protective effect against the opsonizing activity of the surfactant protein A, which implicates a O-glycosylation present in group I alleles of the pilin subunit [126]. A study reported an increased prevalence of group I pilin alleles in isolates from CF patients, compared to non-CF clinical isolates[127].

Both the flagellum and T4P are known to play a role in the formation of biofilm *in vitro*, as mutants of each appendage are impaired at the step of initial attachment and microcolony formation, respectively [128]. These appendages are both important for the binding to lung epithelial cells through different mechanisms: T4P will bind N-glycans at the apical surface of polarized cells while the flagellum will bind to heparan sulfate present at the basolateral side of the cells [129]. Both events will lead to the internalization of *P. aeruginosa* into the cells [129].

On the other hand, using Tn-seq with a transposon mutant library of strain PA14 in an acute pneumonia mice model, Lorenz *et al*. (2019) found that the flagellum and T4P were not required to cause infections [130]. In fact, mutants with transposon insertions in the pili and flagellar biogenesis operons (*pil, flg* and *fli* operons) were enriched (positively selected) in this infection model. Since both pili and flagellar fiber proteins can be recognized by the immune system of the host, the authors suggested that loss of those components might help with immune evasion to prevent bacterial clearance [130,131]. Mucoid variants from CF lungs are non-motile: the lack of functional MucA in these variants will lead to a constant activation of the alternative sigma factor AlgT, which will in turn activate a repressor of flagellar biosynthesis (AmrZ) [75,132]. The flagellum and T4P are still useful during the initial process of infection; however, it is likely that some selective pressure will lead to members of the total bacterial population to lose this trait over time. Part of the selection pressure is mediated through the interaction between the flagellum and NLRC4 inflammasome. Induction of NLRC4 by the flagellum, primarily in alveolar macrophages, impaired *P. aeruginosa* clearance and increased apoptosis/pyroptosis and mortality in a murine model of acute pneumonia [133].

### Lectins

LecA and LecB produced by *P. aeruginosa* have multiple roles in the outcome of lung infections. Single mutants of both lectins are impaired in their ability to adhere to a human lung epithelial cell line, have reduced cytotoxicity and cause reduced permeabilization of the alveolar capillary barrier in mice (without affecting mortality) [134]. The same study showed that carbohydrate lectin inhibitor will counter the effect of their respective lectin by titrating their binding sites. LecA binds to the glycosphingolipids Gs3, triggering phosphorylation of the adaptor protein CrkII by activating the non-receptor tyrosine kinase Abl inside the host’s cell that initiates the internalization of *P. aeruginosa*[135]. Mutation in either *lecA* or *lecB* genes will result in impaired biofilm formation by *P. aeruginosa* [136,137]. A recent study reported that LecB binds to the side chain of Psl and help stabilize the biofilm matrix [138]. LecB is also implicated in the reduction of tissue healing [139,140].

## EXOPRODUCTS AND SECRETION SYSTEMS

### Secretion systems

As discussed above, *P. aeruginosa* expresses five different secretion systems that play a role in virulence (reviewed in [13]). The alkaline protease AprA exported by TSS1 cleaves the γ-subunit of the epithelial sodium channel ENaC *in vitro* on both CF and non-CF lung epithelial cells, leading to changes in the sodium balance near the cells. AprA also cleaves other proteins including transferrin, complements and cytokines, hence facilitating iron uptake and immune evasion [141,142].

The T2SS secretes ToxA, LasA, LasB, and PlcH [13]. The elastase LasB degrades a number of components in both the innate and adaptive immune systems, for instance an absence of LasB secretion impairs the ability of *P. aeruginosa* to degrade pulmonary surfactant protein-A, both *in vitro* and in mouse lungs [143]. By assessing the virulence of T2SS-null mutant deficient in the Xcp secretin, a study highlighted the importance of the effectors secreted by this system: lack of T2SS increased the survival of mice in the context of lung infection [144]. The same study reported that T3SS is responsible for the early killing of mice, while T2SS has a more pronounced impact at later stages of the infection process. This is in agreement with the fact that Xcp and its effectors are positively regulated by the QS [144].

Of the four T3SS effectors injected into the host cells, ExoU contributes the most to the virulence in a mouse model of lung infection, followed by ExoS [145]. ExoS, ExoT and ExoY are all implicated in the systemic dissemination of *P. aeruginosa* from the lung during acute pneumonia [146]. The main cell types injected with ExoU were macrophages during early pneumonia, followed by neutrophils in the latter time points once the immune systems starts building its response to the infection [147]. In a mouse model of acute pneumonia, the transcription of *exoU* was increased as early as 3 hours post-infection, and a delayed expression of this exoenzyme resulted in a significantly reduced bacterial burden [148]. Both ExoU and ExoS are mutually exclusive in the arsenal of *P. aeruginosa*, with the presence of *exoU* in clinical isolates being lower [149]. None of 24 nonclonal isolates from adult CF patients harbor the exoU gene [150]. The contribution of ExoY to *P. aeruginosa* pathogenesis is not as obvious [151]. Loss of *exoY* or its effect alone have little impact on the outcome; however, its overexpression by a high-copy-number plasmid leads to increased and sustained inflammation in a mouse model of lung infection [151]. ExoY is a nucleotidyl cyclase that produces cyclic nucleotides, mainly cUMP, which in turn causes a dysregulation of microtubules, reduction of tissue healing and release of toxic phosphorylated tau proteins [151–153]. Interestingly, the T3SS nanomachine in itself also bear some importance in the pathogenesis of *P. aeruginosa* independent of its known secreted effectors since a mutant lacking the translocator protein PopB was more avirulent than mutants lacking all T3SS effectors in an mouse model of acute pneumonia [154].

The hemolysin ExlA secreted by the T5SS perforates mammalian cell membranes and disrupts the ion balance of the host cells, leading to cell death [155]. ExlA also breaks down the alveolar-capillary barrier and facilitates invasive dissemination of *P. aeruginosa* to other organs [156].

Although most effectors secreted by the T6SS are devoted to interbacterial killing and competition, some of them also modulate host response [17]. Two such T6SS effectors are the phospholipase D PldA and PldB (also known as Tle5 and Tle5B) secreted by the third T6SS complex (H3-T6SS), both can cause lysis of other prokaryotes and trigger pathways that will facilitate *P. aeruginosa* entry into the mammalian host cell [157,158]. Phospholipase D is also associated with persistency since *pldA*-null mutants were severely outcompeted by their wild-type parental strain in a rat chronic lung infection model [159].

### Rhamnolipids

For decades, RLs were only known as the heat-stable extracellular hemolysin of *P. aeruginosa* [160–164]. Then Kownatzki *et al.* found that sputum samples obtained from people with CF colonized with *P*. aeruginosa contained RLs [165]. This was followed by measurement of even higher levels in secretions of a lung removed from a CF patient [166], suggesting that higher concentrations are present in the lower respiratory tract. Eventually, a correlation was reported between elevated levels of RLs and worsened patient’s clinical status [166]. This is not restricted to CF, though, as another study found that patients persistently colonized by *P. aeruginosa* isolates producing high levels of RLs were at increased risk of developing a VAP [167]. Significantly, the importance of RLs for the establishment of an actual infection was demonstrated: a RL-negative mutant was more rapidly cleared from a pulmonary infection mice model compared to the wild type strain [168].

Several properties of RLs makes them versatile virulence factors. RLs inhibit ciliary function of the bronchial epithelium. They alter respiratory epithelial ion transport through reduced sodium absorption and unidirectional chloride fluxes across human bronchial epithelium [164], they interfere with the normal tracheal ciliary function of rabbit tracheal epithelium [169] and slow down the ciliary beat frequency of cultured human airway epithelium [166,170]. RLs also stimulate the release of mucus glycoconjugates from feline trachea and human bronchial mucosa [171,172].

RLs are involved in the early disruption of the integrity of the polarized bronchial epithelia. Normal function of tight junction protein complexes is crucial for lung defense against infections. RLs promote infiltration of *P. aeruginosa* by altering tight junctions in the respiratory epithelium [173–175]. For instance, RLs disrupt transcellular ion transport, as observed by a decrease in amiloride-sensitive short-circuit current across sheep tracheal epithelium [173]. *P. aeruginosa* required the production of RLs to invade respiratory epithelia reconstituted with primary human respiratory cells [175]. Using an air-liquid model of human airway epithelia, Halldorsson *et al*. (2010) found that RLs reduced transepithelial electrical resistance and tight junction rearrangement [176].

RLs have been proposed to solubilize the phospholipids composing the lung surfactant, making them more easily cleavable by phospholipase C (PlcH). The resulting loss of lung surfactant may be responsible for the atelectasis associated with acute and chronic *P. aeruginosa* lung infections. RLs may also intercalate into, and thus perturb, the phosphatidylcholine bilayers, affecting the alveolar surface layer and thus the function of the membrane [177,178].

Not surprisingly, considering they are surfactants, RLs act directly on immune cells. The production of RLs appears important for the initial stage of infection to protect bacteria against the cellular components of the immune system. RLs injected intratracheally into rat lungs inhibited the response of alveolar macrophages [179], and, at higher concentrations, they were cytolytic to human monocyte-derived macrophages [180]. *P. aeruginosa* responds to the presence of polymorphonuclear leukocytes (PMNs), major immune determinants of the CF lungs, by upregulating the production of several virulence factors, including RLs, acting to inactivate cells of the host defense. RLs induce the lysis of PMNs while [168,181,182], at subtoxic levels, they stimulate both chemotaxis and chemokinesis of PMNs [182]. RLs also induce histamine release from mast cells [183], stimulate the production of the inflammatory mediators serotonin and 12-hydroxyeicosatetraenoic acid from human platelets [184], and also stimulate the release of interleukin (IL)-8, GM-CSF, and IL-6 from nasal epithelial cells[185].

Finally, although the direct effect on *in vivo* biofilm has yet to be directly demonstrated, RLs are involved in the regulation of *in vitro* biofilm development, a key virulence function in lung infection, as discussed above. This role of RLs has been reviewed previously [186], and therefore is only briefly discussed here. RLs participate in the regulation of cell-surface hydrophobicity and in the modification of bacterial adhesive interactions [187–189]. They facilitate the initial formation of microcolonies and the differentiation of the biofilm structure [190], by mediating the detachment and dispersion of *P. aeruginosa* cells from the biofilm [187,191] and by maintaining open the channels inside the biofilm [192].

### Pyocyanin

Pyocyanin has been recovered from CF and non-CF bronchiectatic airways at 0.1 mM concentrations [193,194]. Earlier studies have shown that pyocyanin damages ciliated epithelium, interferes with ciliary function, inhibits mucous transport [193], induces neutrophilia and bronchoconstriction in sheep airways [195,196], and decreases mucus velocity in trachea [197,198]. Additionally, the levels of pyocyanin negatively correlate with the lung function of CF patients [194]. Importantly, pyocyanin has been shown to be important for both acute and chronic lung infection [199,200]. Further studies have shown that mouse lungs chronically exposed to pyocyanin develop goblet cell metaplasia and hyperplasia, mucus hypersecretion, fibrosis, emphysema, polarization of an initially T helper cell (Th) 1 toward a Th2 response at chronic stage, with influx of neutrophils, CD4^+^ T cells and activated macrophages [200]. These phenotypes are similar to chronically-diseased airways and to *foxa2*^−/-^ mice [201]. FOXA2 is a major transcriptional regulator of airway mucus homeostasis [62]. Subsequently, it was shown that pyocyanin drives goblet cell metaplasia and hyperplasia and mucus hypersecretion by activating both IL-4/IL-13-STAT6-SPDEF and ROS-EGFR-MEK1/2-ERK1/2 signaling pathways, which converged to inhibit FOXA2 [200,202–205]. Finally, pyocyanin also inhibit FOXA2 expression by ROS/RNS-mediated posttranslational modifications [206].

### IRON ACQUISITION SYSTEMS

Using dual-seq transcriptomics of *P. aeruginosa* infection, Damron *et al*. (2016) highlighted what they called a “battle for iron” [207], Transcript levels of iron acquisition systems in *P. aeruginosa* were found upregulated while the host cells increased its expression of iron sequestering proteins such as ferritin, lactoferrin, ceruloplasmin and haptoglobin [207]. *P. aeruginosa* produces two siderophores – pyoverdine and pyochelin – the former is required for growth in a medium where iron is bound to transferrin, underlining a better capacity for pyoverdine to compete with host iron carriers. The iron-starvation sigma factor PvdS is expressed by *P. aeruginosa* in infected CF lungs [208], and is required for biofilm formation [209]. PvdS also regulates the transcription of genes encoding major virulence factors such as pyoverdine, PrpL and AprA proteases [210], and ToxA [211]. In a mouse model of acute pneumonia, a *P. aeruginosa* mutant deficient in pyoverdine production was more severely attenuated and caused less mortality when compared to a mutant without pyochelin [212]. Additionally, the loss of the *tonB* gene, which encodes a protein that delivers energy to outer membrane transporters important for the uptake of iron, siderophores and other nutrients, renders the mutant bacterium completely avirulent [212,213]. Significantly, *in vivo* pyoverdine production by CF isolates of *P. aeruginosa* correlated to their virulence potential during murine lung infection [214]. However, it is important to note that a sizable portion of CF isolates has also lost their ability to produce pyoverdine, possibly through the emergence of cheaters [215], or adaption toward heme acquisition [216]. Both the ferrous iron uptake system Feo and heme uptake systems Has and Phu also play a minor role in virulence, less important than pyoverdine [212]. The Hxu system has a minor role in heme acquisition when the Has and Phu systems are fully functional, or when other sources of iron are present. However, in the absence of the Has system, *P. aeruginosa* upregulates the expression of Hxu. The contribution of the Hxu system to *P. aeruginosa* virulence remains uncharacterized [46].

## GENETIC TRANSFER, DRUG RESISTANCE AND INCREASED VIRULENCE

The evolutionary process of natural selection, gene loss and acquisition [217,218], natural genetic transformation [219], horizontal gene transfer [220], and emergence of hypermutators during acute VAP and/or chronic CF lung infection can lead to changes in antimicrobial resistance (AMR) and virulence behavior of *P. aeruginosa* [210,221]. The *P. aeruginosa* pan-genome contains a collection of 3,010 putative and plasmids DNA genes obtained by horizontal gene transfer with 5% to 12% encoding AMR and virulence genes [222]. Diversification of the *P. aeruginosa* population in flexible genes indicated that 10,515 (40%) of the 26,420 flexible genes were found in one group of isolates; whereas 85 genes were in more than 90% of the isolates of a single group and were not found in the other groups. Flexible genes (15,905, 60%) present in multiple groups indicated an upper estimate of genes from HGT events. This would suggest that there is a link between virulence factor profiles, AMR and the population structure in *P. aeruginosa.*

Finally, although *P. aeruginosa* virulence determinants that are key to COPD chronic infections have been poorly studied, there are indications they are very similar to those involved in CF [218^218^,^223^].

## EAR INFECTIONS

Middle ear infections (otitis media, OM) are among the most common ear diseases affecting humans. Children, especially those under the age of two, are particularly susceptible to develop OM. Typically, OM is caused by a dysfunction of the eustachian tube connecting the middle ear to the throat. The eustachian tube equalizes the pressure between the outer ear and the middle ear. However, disruption of eustachian tube caused by a common cold, allergies and/or malformations prevents proper drainage, leading to a buildup of middle ear fluid behind the eardrum. The accumulated fluid becomes a culture media supporting the growth of bacteria and viruses that lead to acute OM. If improperly treated, OM can cause considerable morbidity and with extra- and intracranial complications, which seriously compromise the quality of life in patients. OM can be further broadly classified into acute otitis media (AOM) and chronic suppurative otitis media (CSOM). AOM is characterized by tympanic membrane swelling and/or otorrhea caused primarily by *Streptococcus pneumoniae, Haemophilus influenzae*, and *Moraxella catarrhalis*, although occasionally, *P. aeruginosa* is present [224–226].

Despite appropriate therapy, AOM can progress into CSOM associated with ear drum perforation and purulent discharge. Approximately 1% of human population develop CSOM in their lifetime. CSOM is initiated by an episode of acute infection, followed by recurrent or persistent ear discharge through a perforation of the tympanic membrane. *P. aeruginosa* is the most common pathogen involved in CSOM, with a prevalence, depending on studies, between 18% and 67% [227–230]. The pathophysiology of CSOM begins with irritation and subsequent inflammation and edema of the middle ear mucosa. Ongoing inflammation eventually leads to mucosal ulceration and consequent breakdown of the epithelial lining. The cycle of inflammation, ulceration, infection, and granulation tissue formation may continue, destroying surrounding bony margins and ultimately leading to the various complications associated with CSOM, among others, including hearing loss and delayed language development in children, and serious extracranial and intracranial complications.

Additionally, *P. aeruginosa* is also the most common cause of otitis externa (OE), commonly known as “swimmer’s ear”, an infection of the external ear canal [230,231]. A large multicenter study that identified the microorganisms from 2049 ears (children and adults) with acute otitis externa (AOE) revealed that *P. aeruginosa* accounted for 71.3% of all Gram‐negative organisms and for 37.7% of the total number of microbial pathogens recovered from AOE. In some rare instances, AOE can spread to surrounding tissue, including the bones of the jaw and face. This infection is known as malignant otitis externa (OEM), a virulent infection that usually affects elderly with diabetic mellitus, immunosuppressed individuals and advanced age patients. If not diagnosed and treated promptly, OEM can cause facial nerve paralysis with high mortality rate, reported to be up to 53% [232,233].

*P. aeruginosa* is also a major pathogen implicated in cholesteatoma, a destructive and expanding growth consisting of keratinizing squamous epithelium in the middle ear and/or mastoid part of the temporal bone [234]. About 40% of CSOM patients develop cholesteatoma. Bacterial-infected cholesteatomas are more aggressive than uninfected ones. Additionally, recent study suggests that the keratinizing squamous epithelia could be predisposed to infection by human papillomavirus, and DNA belonging to oncogenic HPV16 has been detected in cholesteatoma tissues [235]. Several virulence factors of *P. aeruginosa* have been identified to play a role in the aforementioned ear infections. They are briefly summarized below.

## EXOENZYMES AND SECRETION SYSTEMS

### Proteases

The expression of proteases is regulated by QS [23] as well as iron availability [210]. *P. aeruginosa* proteases participate in CSOM pathogenesis. The activity of two matrix metalloproteinases, elastase and alkaline protease, along with the serine protease neutrophil elastase, were detected in the otorrhea samples from patients with OM and CSOM [236,237]. The importance of elastase and alkaline protease was confirmed by the same authors in a chinchilla model where inhibition of both proteases by the matrix metalloproteinases inhibitor GM 6001 (N-(2(R)-2(hydroxyamido carbonylmethyl)-4-methylpentanoyl)-L-tryptophane methylamide) afforded higher survival rate, less severe facial paralysis, and less vestibular toxicity than animals in other cohorts treated with the gentamicin, gentamicin plus GM 6001, or control groups, although these differences were not statistically significant [238]. Because excess neutrophil elastase causes tissue damage and alters the remodeling process in many clinical conditions including OM, most likely both *P. aeruginosa* and host proteases synergistically contribute to CSOM pathogenesis. Therefore, a protease inhibitor-based therapy targeting all these proteases will likely to be more effective [236].

### T3SS

Although well known to be an important component of *P. aeruginosa* virulence arsenals, virtually nothing is known about the pathogenic role of T3SS during ear infection. Park *et al.* (2017) found that *P. aeruginosa exoU*-positive strains were more readily isolated from CSOM than from respiratory infections and bacteremia. Furthermore, these *exoU*-positive isolates were significantly more resistant to ciprofloxacin and tobramycin, which may explain the chronicity and intractability of CSOM infection [239].

### ToxA

*ToxA is a protein with ADP ribosyltransferase activity that targets host elongation factor 2. It inhibits protein biosynthesis, and in microgram quantities, could penetrate and cause irreversible damage to the inner ears of chinchillas 240 In addition, ToxA induces excessive inflammatory response dominated by polymorphonuclear leukocytes (PMNs) and monocytes/macrophages. Also, epithelial cells often showed signs of metaplasia and hyperplasia, followed by degradation through necrosis and/or apoptosis, resulting in denudation of the submucosal layer 241 Also, a wild-type P. aeruginosa strain (ToxA ^+^) caused significantly more semicircular canal injury and hearing loss than an isogenic ToxA^−^ mutant 242*

### Pyocyanin

As discussed above in the lung section, pyocyanin is redox-active, and ROS and RNS generated by pyocyanin damage proteins, lipids and DNA, and alters host immune signaling [18,62,206]. A major pathophysiological consequence of pyocyanin lies in its ability to cause excessive expression of mucin glycoproteins MUC5AC and MUC5B [62,202,205]. Importantly, pyocyanin has been detected in concentrations up to 2.714 nmoles/g ear effusion of patients suffering from various ear infections, including CSOM [243]. Although MUC5B and MUC5AC play major innate immunity roles against ear infection, excessive induction of these mucins will lead to clogging of inner ear compartments, creating a niche for long term colonization and infection by microbial pathogens [244–246].

## EXOPOLYSACCHARIDES AND BIOFILMS

### Biofilms

Biofilm formation is important in the pathogenesis of CSOM by bacterial pathogens [247–249], although their precise pathogenic mechanisms in the middle ear are not fully understood. *P. aeruginosa* inoculated into cynomolgus monkeys which underwent perforation of the tympanic membrane formed biofilms on the middle ear mucosal surface [250]. Both the ability to produce c-di-GMP and QS were found to be important for persistence and virulence in a chinchilla model of CSOM. However, flagella were dispensable for biofilm persistence [251]. Interestingly, biofilms seem to be dispensable for cholesteatoma pathogenesis. Many *P. aeruginosa* mutants deficient in components required for normal biofilm formation (e.g., FleQ, PilA, AlgD, GalU) caused similar amounts of cholesteatomas growth and bone destruction and deposition as their parental wild-type strain [252,253]. Interestingly, the same authors have found that a T4P mutant (Δ*pilA*) derived from the CSOM strain OPPA8 was attenuated in cholesteatoma pathogenesis in Mongolian gerbils, although the reason for this discrepancy could be caused by strain variation [254]. Even much less is known about the role of polymicrobial biofilms in CSOM, but biofilms composed of *P. aeruginosa* and methicillin-resistant *Staphylococcus aureus* (MRSA) induced a very different gene expression profiles that are involved in immune response, inflammation, signaling, development, and defense; that are not expressed with single biofilms formed by either pathogen [255].

## CELLULAR APPENDAGES AND ADHESINS

### LPS

During *P. aeruginosa*-mediated CSOM, LPS causes mucociliary dysfunction in the middle ear by inducing nitrosylative nitric oxide (NO) and peroxynitrite (ONOO^−^)-mediated pathways [256]. The presence of LPS also positively correlates with several cytokines, including IL1-β, IL-8, TNF-α and RANTES in middle ear effusion aspirate from patients, suggesting that the endotoxin-induced local production of these cytokines plays a role CSOM pathogenesis [257,258]. The involvement of LPS is confirmed in the C3H/HeJ mice that harbor a single amino acid substitution in TLR4, making them insensitive to endotoxin and spontaneously develop CSOM caused by Gram-negative bacteria [259,260]. Humans with polymorphisms and reduced expression of TLRs are more susceptible to CSOM [260,261]. Finally, LPS has been implicated in the bone resorption in mouse model of CSOM and cholesteatomas [262].

### OprF

The outer membrane protein OprF of *P. aeruginosa* activates the host PKC pathway by phosphorylation of PKC‐alpha, leading to the invasion of human middle ear epithelial cells. Induction of PKC can activate the expression of proinflammatory cytokines such as TNF‐α and IL‐1β that can contribute to chronic inflammation [263].

## OCULAR INFECTIONS

The healthy cornea of both eyes is constantly exposed to environmental pollutants, allergens and microbial pathogens, yet maintains immunologically quiescent, thus minimizing excessive damaging proinflammatory responses. However, how the cornea resists microbial infection is not fully understood. The healthy and intact mouse eyes could resist ~10^9^ bacterial cells of either *P. aeruginosa* or *S. aureus*, two most important corneal pathogens, with very few bacteria adhering to the surface and none penetrating the cornea [264,265]. Similar phenomena were observed *ex vivo* using excised intact eyes submerged in bacterial suspension [266], suggesting that components within tear fluid are not required for resistance to bacterial adhesion and infection. Eye infection by *P. aeruginosa* is primarily due to breach of barrier (e.g., scratches on cornea) caused by contact lens immersed in contaminated cleaning solutions, eye trauma and recent ophthalmic surgery. There are more than 140 million contact lens wearers worldwide, with an annualized infection rate between ~2 to ~20 cases per 10,000 wearers. Bacterial keratitis accounts for 90% of all microbial keratitis cases, with *P. aeruginosa* as the most prevalent pathogen, responsible for anywhere between 23% to >50% cases [267,268]. The intrinsic ability of *P. aeruginosa* to resist disinfectants and to adhere to plastic surfaces facilitates its introduction and penetration into the compromised corneal epithelium, gaining further entrance into the corneal stroma. Typically, bacterial keratitis cases can be managed by antibiotic treatment, however, if left untreated, may result in perforation, endophthalmitis and loss of vision. Corneal ulcers caused by *P. aeruginosa* are more severe and difficult to treat, resulting in worse visual impairment [267,269], and may predispose to corneal infection by *Streptococcus pneumoniae* [270,271]. For a comprehensive review on the role of *P. aeruginosa* in contact lens-mediated corneal infection, please refer to Fleiszig *et al.* [272].

As discussed above, intact cornea is resistant to bacterial infection. Most virulence studies in cornea use injury models that bypass the epithelial and basal lamina barriers of cornea and directly deposit bacteria into the stroma [273–275]. However, these models are imprecise because the presence of live/dead *P. aeruginosa* cells and associated pattern recognition receptor ligands (e.g., LPS, pili, flagellum, outer membrane proteins) could trigger host inflammatory responses and mask the pathogenic potential triggered by other virulence factors [273]. The recently developed contact lens-wearing animal models are expected to circumvent the aforementioned challenges [276]. Nevertheless, “scratched” corneal models of infection have helped to identify *P. aeruginosa* virulence factors and host immune responses to infection. Below, we summarize various virulence factors of *P. aeruginosa* that are important for corneal infection.

## CELLULAR APPENDAGES AND ADHESINS

### LPS, T4P and flagella

*P. aeruginosa* initiates infection by binding to receptors on corneal epithelial cells through several “adhesins” including LPS, T4P and flagella. The uptake of *P. aeruginosa* is mediated through the interaction between the outer core of LPS with the cystic fibrosis transmembrane-conductance regulator (CFTR) on lipid rafts, and contributes to persistence, intracellularly survival and disease pathogenesis in the injured murine cornea [277–280]. The LPS-CFTR interaction likely activates PI3K/Akt signaling, leading to the disruption of epithelial cell polarity to promote internalization [281]. In addition to LPS, *P. aeruginosa* uses T4P for adhesion to contact lenses [282] and to sialo-glycoprotein receptor of corneal epithelial cells [283,284]. T4P are important for twitching motility-mediated spread, invasion of corneal epithelial cell multilayers, intracellular movement along collagen fiber, and exiting invaded corneal epithelial cells, as well as *in vivo* virulence [266,275,285–288]. Because flagellin is a ligand for the TLR5, it has been difficult to disentangle the virulence roles of flagella and flagellar-mediated motility from immune response generated during flagellin-TLR5 interactions. Nevertheless, various flagellar components including FlhA (flagellar assembly), FliC (flagellin) and FleQ (regulator flagellar gene expression) are important for adhesion and invasion into the corneal epithelial cells [289,290]. Anti-flagellar antibody protects mouse corneal from infection by *P. aeruginosa *[284].

## EXOENZYMES AND SECRETION SYSTEMS

### T3SS effectors

The ExsA-regulated T3SS effectors (ExoU, ExoS, ExoT, ExoY) are major virulence components of *P. aeruginosa* responsible for cytotoxicity in corneal epithelial cells. Invasive *P. aeruginosa* corneal isolates express ExoS, but not ExoU, with the opposite generally true for cytotoxic isolates [291]. Keratitis isolates tend to favor epidemic clones that express ExoU [292], most likely because they are more resistant to both contact lens disinfection solutions [293], and antimicrobials [294,295]. The activity of RhoGAP domain of ExoT or ExoS alone inhibits the ability of corneal epithelial cells to uptake *P. aeruginosa* bacteria [296]. Additionally, ExoY, which is an adenylate cyclase, is also anti-phagocytic [297]. ExoS and ExoT cause neutrophil apoptosis *via* their ADP ribosyltranferase activity [298], and ADP-ribosylation of Ras by ExoS promotes *P. aeruginosa* survival in the cornea by blocking neutrophil oxidative burst [299]. Also, ExoS causes membrane blebbing within corneal epithelial cells, which are then utilized as a site for bacterial replication and motility [300]. ExoU is a phospholipase that is required for colonization and disease pathogenesis during corneal infection [301]. ExoU promotes traversal in cultured corneal epithelial cells [302], and *P. aeruginosa* survival in a murine scarification model by modulating survival inside phagocytes and infiltration of immune cells called ring infiltration in cornea of infected animals [303].

### Proteases

Liquefactive necrosis of the corneal stroma is a major clinical feature of *P. aeruginosa* keratitis. This bacterium secretes multiple proteases that contribute to the pathogenesis of keratitis [304–306]. Injection of purified LasB elastase and alkaline protease into the corneal stroma results in significant tissue necrosis and keratitis [307,308]. Protease IV contributes to the virulence in keratitis by cleaving multiple host defense proteins including immunoglobulins, complements, antimicrobial peptides and surfactant proteins [309]. The *Pseudomonas aeruginosa* small protease (PASP) degrades collagen, a major structural component of the corneal stroma [310]. When injected into rabbit cornea, PASP destroys the epithelium and cause erosions reaching into the stroma. Additionally, the MucD protease promotes *P. aeruginosa* evasion of immune response during keratitis by inhibiting IL-1β, KC and MIP2 production, neutrophil recruitment and enhancing bacterial survival [311]. Among *P. aeruginosa* proteases, it appears that LasA protease is dispensable for corneal infection [312], most likely due to functional redundancy among proteases expressed by the pathogen.

### ToxA

ToxA disrupts barrier function of corneal epithelial cell monolayers, and exhibits a complementary role with proteases in facilitating *P. aeruginosa* traversal [313]. Direct injection of purified ToxA into the corneal stroma of rabbits caused widespread cell death in a dose-dependent manner [314]. ToxA-deficient mutants were able to induce keratitis as efficiently as their parental wild-type but were more readily cleared by the host, suggesting it plays a role in *P. aeruginosa* persistence during keratitis [315]. Finally, ToxA inhibits synthesis of many proteins including host matrix metalloproteinase 9 (MMP9), while activating other host MMPs in whole rabbit cornea, but the importance of this study is uncertain [304].

## EXOPOLYSACCHARIDES AND BIOFILMS

### Biofilms

*P. aeruginosa* can readily form biofilms on contact lenses worn by rats *in vivo* [276,316], and biofilms represent a significant threat to the cornea from several different perspectives, including increased resistance to antimicrobial factors such as those at the ocular surface. *P. aeruginosa* readily forms biofilms in contact lens storage cases and contact lenses, from which they can disperse and continuously reseed the eyes [317]. As noted above c-di-GMP is an intracellular signaling molecule that coordinates the switch from planktonic to biofilm mode of growth by increasing the production of extracellular matrix components (exopolysaccharides, proteins, extracellular DNA) to form the biofilms [318]. Cyclic-di-GMP binds effector proteins, including FleQ that stimulates the expression of *cdr, pel*, and *psl* genes that control production of adhesins and exopolysaccharides, critical components for biofilm formation [319]. Importantly, FleQ also regulates *P. aeruginosa* invasion of corneal epithelial cells, with contributions in addition to that of flagellin [290].

### IRON ACQUISITION SYSTEMS

Iron acquisition plays an important role in ocular infection. Multiple virulence factors important for eye infection, including Protease IV and ToxA are regulated by iron availability and pyoverdine [320]. Accordingly, a pyoverdine biosynthetic mutant (*pvdE*) exhibits poor invasive ability into corneal epithelial cells, and has attenuated virulence in a murine model of keratitis [321].

## BONE AND PERIPROSTHETIC JOINT INFECTIONS

Orthopedic issues, ranging from bone fractures to joint replacements, are the most common causes of medical care consultation [322], with more than 2.5 million individuals with total hip replacement and 4.7 million individuals with total knee replacement in the United States alone [323]. The demand is expected to increase rapidly with the aging population across the world, and the desire for individuals to stay functional at all ages [324,325]. Periprosthetic joint infection is a devastating complication of total joint arthroplasty and a leading cause of total hip and knee arthroplasty failure [326,327]. Current infection risk reduction approaches to infection include either coating the implant surface with porous or grooved biomaterials to promote soft-tissue ingrowth [328,329], reducing the surface energy to inhibit bacterial adhesion [330–332], or applying layers of antibacterials on the surface to kill the bacteria in vicinity [333–336]. However, implant devices composed of porous materials are associated with a high risk of acute infection in experimental canine and rabbit models [337,338]. Over the longer term, low-energy surfaces become ineffective when a few bacteria attach and eventually form biofilms [339]. Due to elution, tolerance and resistance with recurring use, the efficacy of controlled-release antibiotics will decline overtime [340–342]. Biofilms gradually develop at the abutment-soft tissue interface, leading to recurring and difficult-to-treat infections [343,344]. Superficial infections not only cause pain, erythema, swelling, or purulent discharge at the skin–abutment interface, but could lead to more severe deep infections, and even implant loosing [345].

*P. aeruginosa* is a major pathogen in osteomyelitis and periprosthetic joint infection [346–349]. For example, *P. aeruginosa* causes upward to 75–95% of infections in patients with skull-based osteomyelitis, which typically arise as a complication of OEM (see above ear infection section) with temporal bone involvement [350]. *P. aeruginosa* and other pseudomonads are also responsible for 3–15% of reported cases of diabetic foot osteomyelitis [347]. Although most periprosthetic joint infections are caused by methicillin-sensitive and methicillin-resistant staphylococci [348,351,352], Gram-negative bacterial pathogens maintain a moderate proportion (5%-23%), of these, *P. aeruginosa* is responsible for 25–40% of reported cases [348,351–354]. Also, *P. aeruginosa* is one of the main pathogens (in addition to *S. aureus* and *S. epidermidis*) involved in soft tissue infections among individuals who undergo osseointegration after limb amputation. Finally, *P. aeruginosa* is a significant cause of septic arthritis, one of the most aggressive joint diseases characterized by rapidly progressing joint and cartilage destruction, with a mortality rate of 5–25%^.^ [355,356]. The incidence of *P. aeruginosa*-mediated septic arthritis is higher in immunocompromised patients and intravenous drug abusers. Also, septic arthritis caused by *P. aeruginosa* and other Gram-negative bacteria is associated with higher mortality than those involving Gram-positive organisms (25% vs 6%, respectively), with only 20% of patients with septic arthritis triggered by Gram-negative bacteria regaining joint function [357]. Despite its clinical significance, virtually nothing is known about the molecular pathogenesis and virulence factors required for orthopedic infections. Most of the published studies are clinical case reports and reviews.

## BIOFILMS AND SECRETION SYSTEMS

### Biofilms and T3SS

Recently, Thompson *et al*. (2018) have developed a new mouse model of prosthetic joint infection by using titanium implant, which showed bacterial infection of the bone/joint tissue, biofilm formation on the implants, reactive bone changes, and inflammatory immune cell infiltrates [358]. These authors also demonstrated that a bispecific antibody targeting both T3SS translocon protein PcrV and exopolysaccharide biofilm matrix Psl successfully reduced the burden *P. aeruginosa* in the joint infection. These findings suggest that both T3SS and biofilms participate in the orthopedic infection by *P. aeruginosa*. The importance of T3SS is further corroborated by finding that cartilages of articular joints, which are anatomical sites rarely infected by bacteria, secrete the cartilage-associated antimicrobial factor (CA-AMF), with significantly more antimicrobial activity against *P. aeruginosa* strains with a functional T3SS. The authors proposed that CA-AMF has evolved to selectively target pathogenic bacteria among the beneficial and commensal microflora [359], though the molecular mechanism such antagonism remains not understood. Finally, a recently developed mouse model of septic arthritis infection by *P. aeruginosa* has revealed a protective role by neutrophils against septic arthritis. In contrast, CD4^+^ T-cells play pathogenic role in *P. aeruginosa* mediated septic arthritis [360].

## GASTROINTESTINAL TRACT INFECTIONS

*P. aeruginosa* is usually a transient and not a common pathogen in the gastrointestinal (GI) tract, and isolation of the bacterium in stool is of no clinical significance. However, the presence of *P. aeruginosa* in the proximal intestinal tract is associated with a 70% death rate in critically ill patients, a 3-fold increase over age-matched patients who have negative cultures for this organism [361]. *P. aeruginosa* causes necrotizing enterocolitis in premature infants and in neutropenic cancer patients. For example, *P. aeruginosa* has caused outbreaks of diarrheal diseases in neonatal and pediatric patients [362–364], mostly associated with antibiotic exposure [365,366]. *P. aeruginosa*-mediated neonatal necrotizing enterocolitis are also more frequent in formula-fed newborns [367]. One of the most serious forms of diarrheal diseases caused by *P. aeruginosa* is Shanghai fever, a highly fatal necrotizing enteritis due to rapid onset of septic shock and multiple-organ dysfunction syndrome (MODS) [368]. *P. aeruginosa* also causes infections in individuals who undergo endoscopy. Because endoscopes are made of heat-sensitive materials, they cannot be autoclaved. Endoscopy-related infection by *P. aeruginosa* is typically associated with improper sterilization of reprocessed endoscopes contaminated with gut flora of a patient from previous use [369]. Finally, *P. aeruginosa* is responsible for significant post-surgical infections within the GI tract, and is associated with poor outcome and lack of preoperative oral antibiotic prophylaxis [370–372]. However, very little is known about the virulence mechanisms governing the aforementioned GI-infections.

## QUORUM SENSING

Besides acting as a QS signal, oxo-C_12_-HSL disrupts barrier integrity in the human intestinal epithelial Caco-2 cells by activating p38 and p42/44 kinases, resulting in decreased transepithelial electrical resistance, increased paracellular flux, reduction in the expression and distribution of ZO-1 and occludin, and reorganization of F-actin[373]. Oxo-C_12_-HSL also disrupts epithelial barrier formed by Caco-2 cells by modulating calcium signaling and phosphorylation status of ZO-3 and JAM-A, resulting in reduced expression and distribution of these junction proteins[374]. Oxo-C_12_-HSL also interacts with the IQ-motif-containing GTPase-activating protein IQGAP1, resulting in changes in the phosphorylation status of Rac1 and Cdc42, altering the cytoskeleton network and inducing migration of Caco-2 cells in a dose- and time-dependent manner[375]. Again, these *in vitro* observations need to be interpreted with caution as non-physiological concentrations of oxo-C_12_-HSL were used in the studies using immortalized cell line. A more detailed review of modulation of gut immunity by QS molecules can be found here[376].

## CELLULAR APPENDAGES AND ADHESINS

### LecA

*P. aeruginosa* can use the LecA lectin to adhere to epithelial surfaces. Binding of purified LecA reduces the tight junction permeability formed by Caco-2 cells. Injection of *P. aeruginosa* or LecA-ToxA cocktail directly into the cecum of mice which previously underwent 30% surgical hepatectomy resulted in lethality. Disruption of intestinal tight junction barrier and mouse lethality could be blocked by pretreatment with N-acetyl-D-galactosamine (GalNAc), a receptor for LecA [377,378]. These results indicate that LecA drives the pathogenesis of *P. aeruginosa* in the GI tract by inducing a permeability defect in gut epithelium that allow other virulence factors such as ToxA to penetrate and induce lethality. The expression of LecA is positively regulated by QS, as well as increased by contact with intestinal epithelium and unknown host factors [378]. Interestingly, some of these factors could be the relative concentrations of opioid versus phosphate within the intestinal microenvironment during surgical stress [379].

### T4P

Deletion of the pilin-encoding *pilA* gene did not attenuate adhesions to Caco-2 cells, but rather, blocked both penetration and disruption of Caco-2 cell monolayers. Additionally, the T4P were required for injection of the T3SS effector ExoS into host cells [380]. Finally, a PilA-deficient mutant was attenuated in a silkworm model of intestinal infection. Collectively, the aforementioned studies illustrate a pathogenic process where *P. aeruginosa* binds to intestinal epithelium via LecA and uses T4P to facilitate the delivery of virulence factors that disrupt the epithelial barrier.

## EXOENZYMES AND SECRETION SYSTEMS

The T3SS effector ExoS mediates the penetration of *P. aeruginosa* through the intestinal epithelial cell barrier. After being injected intracellularly, ExoS binds to, and inactivates, the FXYD domain-containing ion transport regulator 3 (FXYD3), resulting in reduction of Na,K-ATPase activity. Inactivation of Na,K-ATPase inhibits the expression of ZO-1 and occludin, leading to a disruption of the tight junction formed by Caco-2 cells, which allows *P. aeruginosa* to transmigrate effectively through the broken epithelial cell barrier [381].

### IRON ACQUISITION SYSTEMS

By comparative genome hybridization analysis of high and low virulence strains, Okuda *et al.* (2012) have identified *P. aeruginosa* genes that are associated with bacterial translocation from the gut epithelial barrier. Among these, the pyoverdine biosynthetic gene *pvdE* is required for penetration through Caco-2 epithelial cell monolayers, and for translocation of *P. aeruginosa* from the gut to the hemolymph in infected silkworms [382]. Analyzing the synergy between PvdE and ExoS revealed that the latter is required for penetration through the intestinal epithelial cell barrier while PvdE is required for virulence and replication in the hemolymph of silkworms, independent of ExoS.

### Virulence genes identified through transcriptomic and mutant library screens

Using the insertion-sequencing (INSeq) approach, Skurnik *et al*. (2013) analyzed the contribution to fitness of all non-essential genes in the chromosome of *P. aeruginosa* strain PA14 during GI tract colonization [383]. Mutants with insertion in almost all known virulence factors as well as genes with unknown functions were identified to be putatively attenuated. Interestingly, 90% of these non-essential genes were required for *in vivo* survival following systemic dissemination during neutropenia [383]. However, the results from an earlier study from the same group seem to contradict these results. Koh *et al*. (2010) compared virulence gene expression from the GI tracts of *P. aeruginosa*-colonized mice to that from the drinking water used to colonize the mice. The expression of genes involved in biofilm formation and T3SS were significantly increased during gut colonization. However, none of the biofilm formation genes were required for GI colonization in the Tn mutant library screen. Similarly, T3SS mutants deficient in ExoS, ExoU, ExoT, and PopB were dispensable for GI colonization and neutropenia-induced dissemination in mice, suggesting that changes in the gene expression during GI colonization is not predictive of an essential role for the gene product in either colonization or overall survival following induction of neutropenia [384].

In a separate study, it was found that monosaccharides present in formula versus those in human milk also appear to modulate the expression of *P. aeruginosa* virulence genes, which may have implication in the pathogenesis of neonatal necrotizing enterocolitis. Arabinose (present in formula), xylose (present in human milk), and galactose (present in both formula and feces from milk-fed infants) caused a significant increase in the expression of virulence genes. In contrast, mannose (present in the feces of milk-fed infants) significantly attenuated the expression of virulence genes [367]. In another study, mucin glycoproteins, which are major components of mucus lining the GI tract and forms the first line of defense against microbial infection, were shown to downregulate the expression of virulence genes that are involved in QS, siderophore biosynthesis and toxin secretion, as well as rapidly disintegrate biofilms in an *in vitro* 3D mucus model [385]. Similar to the aforementioned studies, the importance of *P. aeruginosa* virulence genes would have to be authenticated through single infection in order to clarify their roles in GI infection. However, in a *Caenorhabditis elegans* model of intestinal infection, *P. aeruginosa* seems to have evolved a strategy to exploit host mucin to derive monosaccharides required for a successful infection [386]. Again, these studies underscore the complexity of host–pathogen interactions between *P. aeruginosa* and GI tract, and a caution is in order to properly interpret results from various experiments.

## URINARY TRACT INFECTIONS (UTI)

Urinary tract infections (UTI) are caused by multiple bacterial species and can be community-acquired or nosocomial. Among health care-associated infections, UTI are the most common after surgical site infections and pneumonia. *P. aeruginosa* itself only represents about 5 to 10% of those reported UTI [387]. However, among patients with a complicated UTI, outcomes are usually worst when infected with *P. aeruginosa *[388]. Most *P. aeruginosa*-driven UTI are hospital-acquired through the use of urinary catheters, causing catheter-associated UTI (CAUTI). These infections are characterized by the formation of surface-associated biofilm inside indwelling catheters, leading to an entryway to the urinary tract. From there, *P. aeruginosa* can colonize the bladder and reach the kidneys. (The role of *P. aeruginosa* virulence factors in UTI has been reviewed recently [387], and more extensively but slightly dated[388]). Being the versatile pathogen that it is, *P. aeruginosa* uses multiple-virulence factors to facilitate its survival in the human host. In term of UTI, no specific virulence factor has been described as strictly required for these infections. However, most isolates obtained from UTI are notably proficient at forming biofilms, at producing exoproteases (LasB, AprA) and at causing hemolysis [389].

## EXOPOLYSACCHARIDES AND BIOFILMS

### BIOFILMS

The formation of biofilms appears to be a main feature of *P. aeruginosa* CAUTI, as this phenotype is enhanced in CAUTI isolates compared to those from UTI [390]. The biofilm matrix in CAUTI seems to rely more on extracellular DNA secretion than polysaccharide; this was clearly shown as a Pel- Psl-null double mutant was still capable of colonizing its hosts in a CAUTI mouse model in a manner dependent on extracellular DNA (i.e., sensitive to DNase treatments) [391]. Alginate production is specifically reduced in UTI biofilms, a clear contrast to the biofilms formed in the lungs of CF patients, where isolated strains are often mucoid because they overproduce alginate [392]. Those differences could be linked to the composition of urine: a study combining transcriptomics, proteomics and metabolomics of *P. aeruginosa* PAO1 biofilms grown in artificial urine medium revealed a reduction in alginate synthesis and an increase in the transcription of Fur-regulated genes – including siderophores pyoverdine and pyochelin – compared to biofilms grown in diluted Lysogeny Broth (LB) medium [393]. This study also reported various metabolic adaptations such as reduced denitrification, increased amino acid metabolism and increased expression of QS-regulated virulence factors (proteases LasB and AprA, the lipase LipA and the LecA when grown in artificial urine [393].

### QUORUM SENSING

While QS-null mutants were capable of dissemination through the urinary tract of infected mice, they failed to colonize the organs (bladder and kidney) and cause tissue damage, and were instead rapidly cleared [394–396]. In contrast, this seems to not be the case for CAUTI: another study highlights that QS-null mutants of strain PA14 can colonize the urinary tract as effectively as the wild type in a CAUTI mouse model [397]. The authors also report a reduced expression of QS-related genes when *P. aeruginosa* was grown in PBS-Tryptone combined with human urine; adding urea to the culture medium also reduced the expression of QS-related virulence factors without affecting acyl-homoserine lactone (AHL) production, instead reducing the detection of AHL by their cognate receptors LasR and RhlR [397]. Such a divergence in requirement for QS might depend on the mode of infection, as QS appears to be more important in regulating virulence factors involved in acute vs chronic infections.

### IRON ACQUISITION SYSTEMS

Because urine is recognized as a low-iron environment [398], it is not surprising that siderophore production would be a key virulence determinant of *P. aeruginosa* UTI. The importance of siderophore is highlighted by multiple studies that characterized the *in vitro* production of virulence factors of *P. aeruginosa* isolates from UTI. About 2.5% and 7.5% of 121 *P. aeruginosa* UTI isolates were deficient in pyochelin and pyoverdine production, respectively, when grown under iron-depleted conditions [399]; none of 30 isolates were deficient in pyoverdine production from another study [390]. For the same reason, isolates from UTI often display a proteolytic potential *in vitro *[389]. Proteases such as the LasB and the AprA facilitate iron scavenging by disrupting transferrin – a common iron-bound proteins in urine [142,400].

## EXOENZYMES AND SECRETION SYSTEMS

### Hemolysins

High production of hemolysin might be another factor facilitating the colonization of the urinary tract: Mittal *et al*. measured the activity of multiple virulence factors *in vitro* between urine isolates and assessed their virulence in a mouse model of acute ascending pyelonephritis. They reported a positive correlation between the hemolytic activity and the bacterial load and tissue damage in this model [401]. In a study where virulence determinants of clinical isolates from different infection sites were quantified and compared *in vitro*, isolates from urine had more phospholipase C activity compared to isolates from other infection sites. There were also reduced amounts of ExoS activity produced by urine isolates compared to most other sampling sites, but increased elastase activity compared to blood isolates [402]. In contrast, in another similar study, *P. aeruginosa* isolates from the urinary tract of patients had a pronounced increase in ExoS activity measured *in vitro* compared to tracheal and wound isolates, along with lower ToxA activity. No differences were reported in the activity levels of LasB elastase and phospholipase C between the different groups of isolates [403].

### Pyocyanin

Pyocyanin, the characteristic blue phenazine of *P. aeruginosa*, seems to be less important in the setting of UTI, with an overall lower prevalence of production among isolates from UTI, according to different studies [389,393]. Despite its low reported occurrence, one study described the cytotoxic effects that pyocyanin has on renal tubular epithelial cell when a concentration of pyocyanin ranging between 30 and 60 µM was added to the growth medium: a rise in endoplasmic reticulum vacuolization and proteotoxicity attributed to the strong redox potential of pyocyanin [404].

In summary, to properly colonize the urinary tract of its host, *P. aeruginosa* relies on its capability to form a biofilm and scavenge iron from the scarce environment of the urine. Reliance on QS-regulated virulence factors seems restricted to dissemination of bacteria to upper organs.

## BLOOD-RELATED INFECTIONS

Blood-related infection occurs mostly among patients in intensive-care units and among immunocompromised individuals. Currently, *Pseudomonas* spp. represent a significant cause of hospital-acquired bacteremia, being the third leading cause of Gram-negative bloodstream infections (BSI) [405]. Several studies indicate an increased risk of mortality among patients with *P. aeruginosa* BSI, as compared with the risk for similar patients with other Gram-negative bacteria infections [406]. BSI start when *P. aeruginosa* breaks the epithelial barrier of an affected organ such as lungs, urinary tract or gastrointestinal tract to enter the bloodstream. Central venous catheters can also act as entry points. This often leads to dissemination toward other organs and sepsis, with poorer outcome compared to other bacterial species [406]. Occasionally, this bacterium also causes endocarditis, but occurrence is only significant among intravenous drug users and patients with heart prosthetics [9,407]. Notably, endocarditis can be defined as either left-sided (aortic and mitral valve vegetation) and right-sided (tricuspid valve vegetation), both sides will contain different environmental conditions that will affect the outcome of the infective endocarditis – with left-sided infection linked to poorer outcome [408].

### Prevalence of virulence factors

The high mortality rate of *P. aeruginosa* BSI has been mostly linked to inappropriate initial antimicrobial treatment, typically a consequence of high resistance to antibiotics, instead of the expression of certain virulence factors [409–411]. A study assessed the presence of virulence genes between high mortality rate and low mortality rate of *P. aeruginosa* bloodstream isolates: by comparing patient data with the genomic profiles of the isolated strains, no clear link could be made between the presence of certain virulence factors and the outcome of infection [412]. However, this study was limited because of its small sample size of ten isolates/patients. When comparing the virulence of bloodstream isolates to those from peripheral organs in the *Galleria mellonella* larvae infection model, Hickey *et al*. (2018) remarked that cell-free supernatant from bloodstream isolates killed the larvae much quicker than the peripheral isolates from the same patient [413]. Using proteomics, they identified a higher abundance of the LecA lectin in the supernatant of BSI isolates, among other. Using a mouse bacteremia model, others have pointed at the contribution of pathogenicity island 1 (notably coding for CupD fimbriae and type IVb pili) and pathogenicity island 2 (coding for the potent T3SS effector ExoU) to the virulence of strain UCBPP-PA14 in this infection model [414]. Poor patient outcome was also linked to the presence of ExoU instead of ExoS, presence of these exotoxins being mutually exclusive in *P. aeruginosa* strains [415,416]. The former is generally less prevalent in clinical isolates [417]. In term of lifestyle, isolates from catheter-associated BSI were found to be more motile than those from CAUTI, the latter relying more on biofilm formation [389].

## EXOENZYMES AND SECRETION SYSTEMS

### T3SS and elastase

To reach the bloodstream from an infected organ, *P. aeruginosa* needs to traverse the epithelial barrier of the organ and the endothelium of the adjacent blood vessel. To that end, *P. aeruginosa* will use T3SS and its effectors to induce tissue damage [15]. ExoU is a potent toxin with a intracellular phospholipase activity akin to the human phospholipase A2 that will induce plasma membrane disruption upon translocation into the cytosol of host’s epithelial cells [418]. An *exoU*-positive genotype is an independent predictor of early mortality in *P. aeruginosa* BSI [415]. ExoS and ExoT are both proteins containing GTPase-activating domain and ADPRT domain inhibiting the actin cytoskeleton and leading to cell death [15]. The role of ExoS in the dissemination from the alveolar epithelium through the endothelium of blood vessels into the blood stream is already reviewed elsewhere [419]. In short, ExoS will partner with the LasB elastase: the former will cause damage to the epithelial cells while the latter – given access to the endothelium of the blood vessel – will cleave vascular endothelium cadherin and disrupt anchor between neighboring cells by damaging tight junction-associated proteins. Apart from T3SS-mediated toxicity, other factors contribute to the damage that *P. aeruginosa* can cause to the tissue.

## EXOPOLYSACCHARIDES AND BIOFILMS

### Alginate

In the case of endocarditis, only a few studies report the role of certain virulence factors. By comparing the virulence of a mucoid *P. aeruginosa* strain and a non-mucoid variant in a rabbit endocarditis model, Bayer *et al*. (1992) demonstrated that the mucoid strain resisted antibiotic treatment and killing by polymorphonuclear leukocytes much better than its non-mucoid counterpart, resistance that was abolished with the injection of alginase[420].

## IRON ACQUISITION SYSTEM

Another study reports that the alternative sigma factor PvdS plays a role in the development of aortic valve infected vegetation (left-side), as a *pvdS*- mutant reached lower bacterial loads and induced less metastatic infections than the wild type, a difference that was not observed during infection of the tricuspid valve (right-side) [421]. It is likely that this results from the difference in oxygen levels between the two sides of the heart, as the oxygen levels in the aorta are higher. They also demonstrated *in vitro* PvdS expression is higher when *P. aeruginosa* is grown under aerobic vs microaerobic conditions [421].

In summary, it is clear from numerous studies that motility, lectins and T3SS are involved in BSI. Virulence factors regulated by PvdS and alginate play a role in the establishment of infective endocarditis.

## BRAIN

*P. aeruginosa* is a rare causative agent of bacterial meningitis and ventriculitis. Such pathologies happen in the most cases following neurosurgeries, such as shunt insertions and external ventricular cerebrospinal fluid drainages, which serve as a direct mode of entry for this pathogen [422]. As is the case for most *P. aeruginosa* infections, poor outcome is tightly linked to the antibiotic resistance profile of the infecting strain and the timely administration of the antibiotic therapy, with intrathecal injection showing the best outcome with adequate antibiotics [11,422].

## EXOENZYMES AND SECRETION SYSTEMS

### T3SS

There have been association between neurodegenerative diseases and the presence of *P. aeruginosa*. During acute pneumonia, patients were found to have an increased amount of amyloid β and tau protein in their cerebrospinal fluid and bronchoalveolar lavages [423]. Reduction of amyloid β 42 and increase in phosphorylated tau proteins in the cerebrospinal fluid are biomarkers of the development of neurological disorders such as dementia and Alzheimer’s disease [424]. Both exoenzymes ExoU and ExoY induce phosphorylation of tau protein, each with a distinct mechanism [153]. ExoY acts as an adenylate cyclase that causes the phosphorylation of tau through the AMPc-dependent protein kinase A and its release from microtubules [425–427]. ExoU is a phospholipase that releases arachidonic acid after its enzymatic cleavage of a phospholipid, which was shown to induce polymerization of tau *in vitro* [428,429]. Additional research is needed to further characterized the role of various virulence factors in this organ.

## SKIN AND SOFT TISSUE INFECTIONS

Our skin serves as the first barrier against the entry of external pathogens. However, once injured – through heavy burns or other wounds – bacterial colonization becomes possible. *P. aeruginosa* causes a variety of skin and soft tissue infections ranging from benign to life-threatening, and it is an especially common pathogen of skin in burn wound, surgical wounds and diabetic foot ulcers. While burn wound infections are often considered acute and lead to rapid dissemination into the bloodstream and lethal sepsis, surgical wounds and ulcer-related infections are considered chronic infections that persist for longer time [430].

## WOUND AND BURN WOUND INFECTIONS

### EXOPOLYSACCHARIDES AND BIOFILMS

## Biofilms

*P. aeruginosa* is a prevalent pathogen in burn wound infections, accounting for about 30% of reported cases [431]. An important factor for chronic *P. aeruginosa* infection of the skin is the formation of a biofilm. Using a model of full thickness scald burn wound rat model, Brandenburg *et al*. (2019) observed a bacterial biofilm that is produced on the burned skin by *P. aeruginosa* [432]. They quantified an elevated expression of *alg* and *pel* from this biofilm, which indicates to importance of both exopolysaccharides in the composition of skin biofilm.

### QUORUM SENSING

Mutants in the main components of the *las* and *rhl* QS systems were much less virulent in an acute burn mouse model [433]. Interestingly, loss of these QS systems does not abolish biofilm formation on the skin of burned mice, nor colonization around blood vessels, as demonstrated with electron and confocal microscopy [434].

## EXOENZYMES AND SECRETION SYSTEMS

### Volatile metabolites

Wounds heavily infected with *P. aeruginosa* have a distinctive odor resembling grape. This odor is caused by the volatile metabolite 2-aminoacetophenone [435], that is used by *P. aeruginosa* to promote its chronic infection phenotype [436], including through immunomodulation [437].

### Pyocyanin

It seems that *P. aeruginosa* can sense the damaged state of wounds, as a recent study demonstrated that growth with homogenized fascia – the collagen-rich connective tissue that envelops muscles – significantly induces the production of pyocyanin and the transcription of pyochelin biosynthesis genes [438]. Another study quantified the amount of pyocyanin from burn wound exudates to assess its importance in tissue damage. The levels of pyocyanin reached up to 5.3 µg per grams of exudates [439]. When similar concentrations of pyocyanin were applied to human fibroblasts, it caused a reduction in tissue repair and cell doubling rate.

## CELLULAR APPENDAGES AND ADHESINS

### Flagella and T4P

Turner *et al*. (2014) used Tn-Seq to assess the fitness determinants in acute burn and chronic wound infection of a mouse model [430]. They identified flagellar motility and chemotaxis as important for the fitness of *P. aeruginosa* PAO1 in the burn wound setting, while T3SS and T6SS were important factors in the chronic wound model. T4P and Psl production were required in both infection types to maintain a high fitness. Using RNA-seq, they also highlighted that iron acquisition gene, T2SS and T3SS are upregulated *in vivo* – in both acute and chronic models – compared to lab-grown planktonic bacteria. LPS modification clusters were downregulated in both infection settings, especially in the chronic wound model [430]. In addition, metabolic transcription profiles indicated that growth in wounds relies on long chain fatty acids metabolism, mutants incapable of catabolizing such carbon source having a virulence defect in both mouse models. This study highlighted the physiological differences that occur depending of the infection caused by *P. aeruginosa*. The importance of flagella and pili assembly genes had previously been demonstrated in a burn wound mouse model, where mutants unable to form either (or both) flagellum and T4P had reduced lethality and dissemination to other organs [440,441]. Indeed, the flagellum has been implicated as a major virulence determinant in normal epidermis tissue invasion: flagellin-null mutants – but not T2SS or T3SS mutants – were unable to penetrate and invade the epithelial layer during infection of reconstructed human epithelium. This was confirmed in mouse model of sub-cutaneous infection [442].

### Virulence genes revealed by transcriptomic approach

Because bloodstream infections often occur after a severe burn trauma, Kruczek *et al*. (2016) compared transcription profile of *P. aeruginosa* PA14 between growth in whole blood from a healthy volunteer and from three severe burn victims [443]. Iron acquisition genes were amongst the most affected in their regulation: transcription of ferrous uptake genes and pyochelin synthesis genes, but not pyoverdine, was markedly increased. On the other hand, QS-regulated genes were under-expressed in whole blood from burn victims compared to the healthy individual. Pilin and fimbriae genes were downregulated, but flagellum genes were upregulated, suggesting activation of flagellar motility by the blood of burned patients compared to the blood of healthy volunteers. It appears from these studies that QS, iron uptake and motility systems play major roles in the unfolding of burned skin infections.

Another study used RNA-seq to evaluate changes in the transcription profile of *P. aeruginosa* PAO1 when grown in burn wound exudates [444]. Under these culture conditions, iron-acquisition and heme-import clusters were upregulated. In addition, T6SS was upregulated while T3SS was downregulated. The transcription of genes involved in QS and its related virulence factors was activated very early during growth in burn wound exudates. By comparing the chemical composition of the medium before and after the growth of *P. aeruginosa*, the authors noticed that triglycerides, cholesterol and lactate were consumed first [444].

## CHRONIC FOOT ULCERS

### BIOFILMS, EXOENZYMES AND SECRETION SYSTEM

Prevention of wound healing is a trait of chronic skin infection. Biofilm formation delays tissue repair of chronic diabetic foot ulcers, venous leg ulcers and pressure ulcers [445,446]. Infecting a biopsy-punch wound of diabetic mice with biofilm-grown *P. aeruginosa* delayed wound healing from 4 weeks to 6–8 weeks compared to uninfected wounds [447]. Both LasB elastase and protease IV reduced wound repair through the inhibition of cell migration and angiogenesis [448]. Both proteases were better produced by high biofilm-forming strains isolated from diabetic foot ulcers compared to moderate or low biofilm-forming isolates from the same infection site. This impact on wound healing was demonstrated by treating mouse wounds with each purified proteases [448]. Jacobsen *et al*. (2012) also demonstrated that *P. aeruginosa* wildtype supernatant – but not supernatant from double *lasR- rhlR*- QS mutant – impaired cell migration of keratinocytes and fibroblasts [449]. The T3SS apparatus also plays a role in these infections. T3SS was demonstrated to contribute to the delay in wound healing in a diabetic mouse model [450].

## FOLLICULITIS

### BIOFILMS, SECRETION SYSTEM AND CELLULAR APPENDAGES

Finally, maybe the most common *P. aeruginosa* infection is a skin rash: this pathogen causes hot tub dermatitis, a condition characterized by a folliculitis that frequently afflicts users of pools and hot tubs [451]. This community-acquired skin infection results from the colonization of hair follicles following exposure to contained, contaminated water (e.g., whirlpools, swimming pools, water slides, bathtubs). Normally benign, such infections can evolve in immunocompromised individuals into severe ecthyma gangrenosum, expressed as gangrenous ulcers with erythematous borders [452]. There is close to no information on the virulence factors required to induce folliculitis and dermatitis. Intriguingly, *P. aeruginosa* serotype O11 was shown to be very prevalent in these types of skin infections [453,454]. Although they were predominant in whirlpool water, these isolates were not more resistant to chlorine [454]. However, we know that the ability to form stress-resistant biofilms is a feature required for the colonization of plumbing fixtures in whirlpool recirculation systems [455]. Furthermore, *P. aeruginosa* isolates secreting ExoU are frequently serotyped as O11 and typically isolated from acute infections [456]. Maybe not surprisingly, a correlation was found between the O11 serotype, positivity for *exoU* and novel *pilA* type in a collection of corneal ulcer isolates with good twitching motility [457]. In a sub-cutaneous infection model, exoenzyme ExoU was shown to be required to induce formation of abscesses in healthy mice, but not in neutropenic mice. The authors suggested that inhibition of neutrophils through the T3SS might be the cornerstone of bacterial growth and tissue invasion in skin diseases [458].

## FUTURE PERSPECTIVES

Tremendous amount of advances have been achieved in the understanding of *P. aeruginosa* pathogenesis over the last few decades. What are the opportunities and challenges lying ahead in the age of “omics”? Some of these major challenges, with a focus on specific tissues and organs are: 1) direct *in vivo* analysis of the expression of key virulence factors during infection of specific organs; 2) direct analysis of the *P. aeruginosa* virulence process and its interactions with host cells through the initiation of an infection to resolution of the disease in tissues and organs. These challenges can now be addressed by using *in vivo* and single-cell transcriptomics, and by three-dimensional bioprinting of so-called primitive human lungs combined with comparative transcriptomics profiling of healthy versus diseased primitive lung constructs.

Direct *in* *vivo P. aeruginosa* transcriptomics has been attempted using different strategies and identified hundreds of genes specifically expressed during infection but not *in vitro *[459]. For example, Conforth *et al.*(2018) compared the *P. aeruginosa* gene expression patterns of clinical samples analyzed directly with *apriori* no growth *in vitro* from CF sputum, chronic wound infections, and burned wound infections, or *in vivo* mouse models of acute pneumonia, burn infections or surgical wound infections. These authors found that transcriptomic profiles of *P. aeruginosa* genes obtained from murine surgical wound model was more closely resembled those of human infections. In contrast, the transcriptomic profiles of *P. aeruginosa* genes in murine models of acute pneumonia and burn sepsis more resembled *in vitro* gene expression profiles. Additionally, some of the genes were not so-called typical virulence factors but instead *lexA* (SOS stress response), *mex* (efflux pumps), *fts*H (protease), *ZnuABC* (bacterial growth), *faoAB* (long-chain fatty-chain metabolism), and a collection of hypothetical genes [460].

The analysis of within-host evolution of *P. aeruginosa* and other bacterial pathogens is now possible by using single-cell transcriptomics, and may lead to the prediction of clinical progression of bacterial infections and options in therapy [461]. For example, single-cell RNA sequencing was used to examine immune cell dysfunction in the pathogenesis of bacterial pneumonia-derived sepsis [462]. A combined strategy could be used by following gene expression of *P. aeruginosa* lung infections using micro-biopsied lung samples and single bacterial cells isolated from tissues by laser microdissection. Although challenging, these technologies are constantly improving and excellent trajectory is being made in single-cell genomics and transcriptome profiling, which will offer different insights into bacterial pathogenesis over metagenomic approaches [426].

It is well known that, in many cases, data acquired through animal models do not faithfully recapitulate various human lung pathologies and diseases, including CF [463]. Due to major genotypic, phenotypic and metabolic differences, novel therapeutics derived from animal models do not always translate into efficacy in humans [464]. In addition, classical cell culture approaches lack the complexity and tissue-like structures relevant to detailed biological analysis. Recent progress and feasibility has been obtained in development of organs-on-a-chip [465], and new technologies that pave the way for 3D bioprinting of complex tissues, including human skin, cartilage, bone, and the air-blood barrier [466,467]. Even though human lungs contain a repertoire of more than 60 specialized cells, we have initiated the next level of lung 3D bioprinting and transcriptomics profiling by focusing on lung epithelial and alveolar cells, leading to a systematic scaling-up approach for additional lung cells and vascularized constructs (Kukavica-Ibrulj I, Potvin, M. and Levesque R. C., unpublished). Another area of advances is in the development of organoids derived from human pluripotent stem cells to model organ development and disease pathogenesis, including in the context of lung–pathogen interactions [468–470]. Combining these new advances with newly developed high-definition spatial transcriptomics [471,472] that can be coupled with single-cell analysis [473,474] to perform *in situ* profiling of infected organs and tissues will be highly desirable. Additionally, implementation of the novel integrative multi-omics approaches to acquire, analyze, and model multimodal data will facilitate disentanglement of the influences of genetic, environment, immune status, (post)transcriptional, (post)translational, and metabolic processes driving the initiation and resolution of disease pathogenesis [475,476].

Finally, antibodies against single *P. aeruginosa* epitopes have been examined for a long time, but without satisfactory protective efficacy. In recent years, there has been an effort to develop bispecific monoclonal antibody (mAb) that simultaneously targets two surface epitopes on *P. aeruginosa*. Significantly, when administered prophylactically or post-infection, the bispecific mAb targeting a component of the T3SS translocon PcrV and the biofilm exopolysaccharide Psl protected against mouse models of lethal acute pneumonia [477], and joint infection [358]. Additionally, when administered either prophylactically or post-infection, subtherapeutic dose of bispecific mAb synergized with subtherapeutic doses of the antibiotics to protect mice in a lethal pneumonia model [477]. Importantly, similar efficacy was achieved by using a DNA delivery method, which circumvents the hurdles of traditional mAb delivery through direct, *in vivo* immunoglobulin production [478]. Finally, in contrast to the broad-spectrum antibiotic approach, pathogen-specific targeting with therapeutic bispecific mAbs would leave the beneficial microbiota intact.

Overall, the aforementioned approaches will expand the current frontiers in our understanding of *P. aeruginosa* pathogenic processes and host responses in tissues and organs.

## CONCLUSIONS

In summary, many years of dedicated research has significantly advanced our understanding of *P*. aeruginosa pathogenesis, especially in the context of chronic lung infections in CF (Figure 1). However, much is still to be revealed about the virulence mechanisms in the rest of organ systems. Because of its ubiquitous presence in the environment, metabolic versatility, high intrinsic resistance to different types of chemotherapeutic agents and antibiotics, and large repertoire of virulence arsenals, *P. aeruginosa* is a very difficult pathogen to control and will likely persist as a significant clinical challenge to modern medicine for many years to come. Therefore, more in-depth understanding of *Pseudomonas* virulence regulation, development of novel antimicrobial approaches, better antibiotic stewardship, as well as vaccine development are warranted. Development of novel animal models that better mimic *P. aeruginosa* infectious diseases of humans as well as rapid advances in the new “omics” technologies will greatly advance these challenges. Particularly, major increase in the understanding in the molecular and cellular events governing free living planktonic mode of growth to biofilm formation, and transition from acute infection to chronic colonization may drive novel anti-*Pseudomonas* approaches.

## Data Availability

Data sharing not applicable – no new data generated.
